# Different hemispheric specialization for face/word recognition: A high‐density ERP study with hemifield visual stimulation

**DOI:** 10.1002/brb3.1649

**Published:** 2020-05-04

**Authors:** Naomi Takamiya, Toshihiko Maekawa, Takao Yamasaki, Katsuya Ogata, Emi Yamada, Mutsuhide Tanaka, Shozo Tobimatsu

**Affiliations:** ^1^ Department of Clinical Neurophysiology Graduate School of Medical Sciences Neurological Institute Kyushu University Fukuoka Japan; ^2^ Department of Physical Therapy Faculty of Health and Welfare Prefectural University of Hiroshima Hiroshima Japan

**Keywords:** Event related potentials, face and word recognition, hemifield visual stimulation, subliminal and supraliminal perception

## Abstract

**Introduction:**

The right fusiform face area (FFA) is important for face recognition, whereas the left visual word fusiform area (VWFA) is critical for word processing. Nevertheless, the early stages of unconscious and conscious face and word processing have not been studied systematically.

**Materials and Methods:**

To explore hemispheric differences for face and word recognition, we manipulated the visual field (left vs. right) and stimulus duration (subliminal [17 ms] versus supraliminal [300 ms]). We recorded P100 and N170 peaks with high‐density ERPs in response to faces/objects or Japanese words/scrambled words in 18 healthy young subjects.

**Results:**

Contralateral P100 was larger than ipsilateral P100 for all stimulus types in the supraliminal, but not subliminal condition. The face‐ and word‐N170s were not evoked in the subliminal condition. The N170 amplitude for the supraliminal face stimuli was significantly larger than that for the objects, and right hemispheric specialization was found for face recognition, irrespective of stimulus visual hemifield. Conversely, the supraliminal word‐N170 amplitude was not significantly modulated by stimulus type, visual field, or hemisphere.

**Conclusions:**

These results suggest that visual awareness is crucial for face and word recognition. Our study using hemifield stimulus presentation further demonstrates the robust right FFA for face recognition but not the left VWFA for word recognition in the Japanese brain.

## Graphical abstract

We systematically investigated ERP responses to facial and word stimuli using visual half‐region stimuli in Japanese adults who routinely use Kanji words. Our results suggest that visual recognition is essential for face and word recognition. Furthermore, our results provide electrophysiological evidence of hemispheric specialization in the fusiform gyrus of the Japanese brain, depending on the category of stimulation.

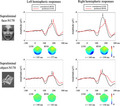

## 1 INTRODUCTION

The human brain exhibits hemispheric specialized. The most notable examples of this specialization are for face and word recognition: The former is lateralized to the right hemisphere (RH; Davies‐Thompson, Johnston, Tashakkor, Pancaroglu, & Barton, 2016; Kanwisher, McDermott, & Chun, 1997), whereas the latter is preferentially processed in the left hemisphere (LH; Cohen & Dehaene, 2004; Dehaene, Le Clec, Poline, Le Bihan, & Cohen, 2002). Face recognition is one of the most important social functions, which are indispensable for survival. Furthermore, not only humans but also animals have this inherent ability (Gross, Rocha‐Miranda, & Bender, 1972). Humans also acquire visual word recognition ability through the influences of culture and education, and spontaneous brain development (Dehaene et al., 2010). The key issue addressed by this study is the profile of lateralization for face and word recognition using high‐density ERPs. More specifically, we focused on the early stages of unconsciousness or conscious face and word processing when visual stimuli were presented in each hemifield.

The human visual system is characterized by parallel and hierarchical processing via the ventral and dorsal streams (Livingstone & Hubel, 1988), with the ventral stream, from the primary visual cortex (V1) to the fusiform gyrus, specialized for face and word recognition. Basic visual features are processed at V1, while complex features of faces and words are mainly processed at the right fusiform face area (FFA) and the left visual word form area (VWFA; Cohen et al., 2000; Issa, Rosenberg, & Husson, 2008; Kanwisher, 2000; Kanwisher, Woods, Iacoboni, & Mazziotta, 1997), respectively. As electrophysiological markers, the P100 and N170 peaks have been extensively studied to explore face and word processing in humans. The P100 component at the occipital area is considered to be an indicator of V1 activity, whereas the N170 component at the occipitotemporal area is thought to reflect the function of the fusiform gyrus (i.e., FFA and VWFA). Although N170s for face and visual word stimuli appear in the same general area, within the same time range, they have different neurophysiological characteristics (Bentin, Allison, Puce, Perez, & McCarthy, 1996; Bentin, Mouchetant‐Rostaing, Giard, Echallier, & Pernier, 1999; Cohen, Jobert, Le Bihan, & Dehaene, 2004; Gauthier, Skudlarski, Gore, & Anderson, 2000). Here, we refer to the N170 evoked by face stimuli as the face‐N170, and that evoked by visual word stimuli as the word‐N170. Several reports have suggested that FFA is located in the right fusiform gyrus, while VWFA is observed in the left fusiform gyrus. In other words, the face‐ and word‐N170s represent functional asymmetry of the cerebral hemispheres (Horie, Yamasaki, Okamoto, Nakashima, et al., 2012; Rossion, Joyce, Cottrell, & Tarr, 2003; Selpien et al., 2015).

The Japanese reading system comprises Hiragana and Katakana for phonological processing and Kanji for lexical semantic processing. An ERP study using full‐field Kanji stimulation showed that Kanji stimulation was more left‐lateralized in native Japanese readers than in native English readers (Maurer, Zevin, & McCandliss, 2008). Additionally, the priming effect, which facilitates more quickly and accurately behavioral visual recognition by visual repetition of words and reduces activation in VWFA, is more pronounced with Kanji than Hiragana (Nakamura, Dehaene, Jobert, Le Bihan, & Kouider, 2005). Therefore, Japanese Kanji is useful for understanding visual word processing.

There have been multiple ERP studies that applied visual hemifield stimulation (Cohen et al., 2000; Honda, Watanabe, Nakamura, Miki, & Kakigi, 2007; Nemrodov, Harpaz, Javitt, & Lavidor, 2011; Towler & Eimer, 2015). In contrast to the ERP studies with full‐field stimulation, previous hemifield studies have shown inconsistent results regarding human hemispheric specialization for face and word recognition. For example, a previous ERP study (Honda et al., 2007) reported that upright and inverted face stimuli presented in the left visual hemifield (LVH) evoked a large face‐N170 in the RH. On the contrary, another study (Towler & Eimer, 2015) demonstrated that there was no RH superiority when face and house stimuli were binocularly presented in the LVH and right visual hemifield (RVH), respectively. A word‐N170 was evoked strictly from word stimuli in the visual hemifield, regardless of stimulation side, in the left inferior temporal area including VWFA (Cohen et al., 2000). However, another study (Nemrodov et al., 2011) showed that the word‐N170s from word and nonword stimuli presented in the visual hemifield did not show left hemispheric specialization but did show contralateral predominance in both LH and RH. In a study of the N400, an ERP component of semantic processing, a hemispheric difference was also found using hemifield word stimulation (Atchley & Kwasny, 2003). Of note, neuroimaging studies have demonstrated that FFA was more activated by face stimuli presented in the LVH than by those in the RVH (Hemond, Kanwisher, & Op de Beeck, 2007), and vice versa for VWFA with word stimuli (Cohen et al., 2000).

To explore the early stages of face/word processing, it is necessary to quantitatively manipulate the level of stimulus recognizability. Here, we adopted perceptual masking to differentiate between automatic and controlled (top‐down) processes, and to interrupt higher processing and prevent the overt recognition of stimuli, based on results in our previous study (Mitsudo, Kamio, Goto, Nakashima, & Tobimatsu, 2011). In that study, we found that visual stimuli presented for 20 ms were unrecognizable (subliminal condition) but that the P100 amplitude was augmented for faces but not objects. However, a clear N170 was not evoked under the subliminal condition. Conversely, visual stimuli presented for 300 ms were easily recognized (supraliminal condition) and evoked a distinct N170; the P100 amplitude was increased compared with that in the subliminal condition. However, to the best of our knowledge, there have been no reports that systematically explored the effect of visual hemifield on ERP responses (P100 and N170) for faces and words in the same subjects. Furthermore, in the subliminal condition with full‐field stimulation, P100 amplitudes to face stimuli were significantly larger than those to objects (we named it the subliminal face effect; Mitsudo et al., 2011). Hence, faces and objects of which the observer is unaware would be processed in a different way at V1 before face‐specific processing occurs within the FFA (Fujita et al., 2013; Mitsudo et al., 2011). However, it is still not known whether this phenomenon is observed during visual hemifield stimulation. Full‐field and hemifield stimulation initiate mainly visual perception from the fovea and parafovea, respectively. The former is important for fine visual perception, whereas the latter can be used to determine the gist of a scene, for a categorization judgment, although with reduced sensitivity and speed compared with foveal vision (Thibaut, Tran, Szaffarczyk, & Muriel, 2014). In face recognition, it is thought that parafoveal perception is important for detecting the warning signs (i.e., fearful face) of potentially threatening situations (Rigoulot et al., 2011). In word recognition, visual fixation on the initial letters of a word, which consists of a string from left to right (i.e., English words), makes the longer part of the word fall in RVH. Conversely, Japanese people read not only from left to right but also from top to bottom, and Kanji does not comprise letter‐strings in the first place. Therefore, it is likely that Kanji and alphabetical words are processed differently when presented either in the central visual field or hemifield.

Based on these earlier observations, the purpose of this study was to clarify the functional brain differences in face and visual word recognition, using ERPs with hemifield stimulation under subliminal and supraliminal conditions. For RVH stimuli, which are primarily perceived by the left visual cortex, this process relies exclusively on pathways confined to the LH and vice versa. We systematically investigated hemispheric superiority for face/word recognition in early visual processing using hemifield stimulation. Our working hypotheses were as follows. First, the P100 response is differentially modulated by stimulus type under the subliminal condition. Even if a face is invisible, it is recognized as a face due to rich low‐spatial‐frequency (low‐SF) information (Nakashima et al., 2008). Conversely, a close link between Kanji and high‐spatial‐frequency (high‐SF) information (Horie, Yamasaki, Okamoto, Kan, et al., 2012; Horie, Yamasaki, Okamoto, Nakashima, et al., 2012) suggests that Japanese word recognition can be difficult under subliminal conditions. Moreover, as far as we know, hemifield stimulation has not been applied to Japanese word stimuli. We assume that there are differential effects of hemifield stimulation on the face‐/word‐P100 in the subliminal condition. Second, the P100 and N170 responses to face and word stimuli show different results in the supraliminal condition. The P100 reflects the initial visual processing at the primary visual cortex. We investigate whether the P100 is influenced by the stimulus type and visual field. When face/word stimuli are presented in the central visual field, RH predominance of face‐N170 and LH predominance of word‐N170 are observed (Rossion et al., 2003). However, this hemispheric predominance has not been recognized in the Kanji‐N170. Therefore, we manipulated the visual field (left vs. right) and stimulus duration (subliminal [17 ms] versus supraliminal [300 ms]).

## 2 MATERIALS AND METHODS

### 2.1 Selection of participants

Japanese writing is peculiar in that it has three different sets of characters: Kanji, Hiragana, and Katakana. Among them, Kanji consists of ideographs, like Chinese characters, which represent whole or partial word meanings. Hiragana and Katakana are phonogram‐like alphabets. Because of this idiosyncrasy, only individuals who had native familiarity with the Japanese language were eligible to be subjects. Eighteen healthy participants (nine females, 21–27 years old) who self‐reported right‐handedness were recruited; all were university students or college graduates. All participants had normal or corrected‐to‐normal vision. None had a history of neurological or psychiatric disorders. All provided their written informed consent for the study, prior to its commencement. The experimental procedures complied with the Declaration of Helsinki and were approved by the ethics committee of the Graduate School of Medical Sciences, Kyushu University.

### 2.2 Stimuli and apparatus

**FIGURE 1:**
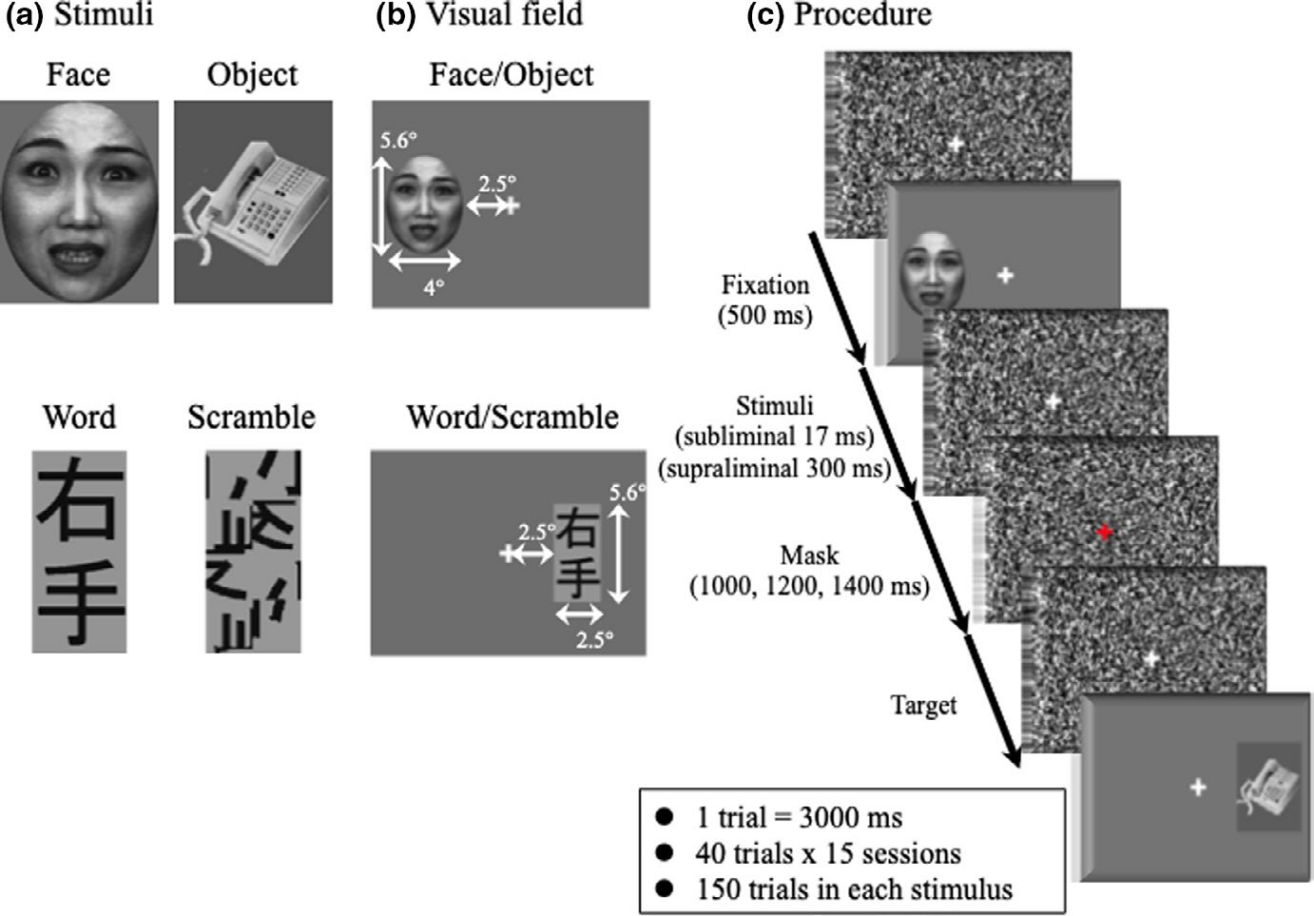
(a) Shown are representative examples of the fearful‐face, object, word (Japanese Kanji), and scrambled‐word stimuli used in this experiment. (b) Visual stimuli were presented either in the right visual hemifield (RVH) or left visual hemifield (LVH), in a pseudo‐random order. The viewing angle from the fixation cross to the inner side of each stimulus was 2.5° horizontally. (c) Experimental procedure. The visual stimuli were followed by presentation of the central fixation cross, on a pattern mask, for 500 ms. Stimuli were presented for two different durations: subliminal (17 ms) and supraliminal (300 ms). Then, a pattern mask was presented for 1,000, 1,200, and 1,400 ms, chosen in a pseudo‐random order. When the fixation cross changed its color, participants clicked a mouse as quickly as possible. Note that the Kanji characters appearing in A mean “right hand”

We used fearful faces and Japanese Kanji words as the visual stimuli (Figure 1a). We chose these stimuli based on the facts that the FFA was activated in early visual processing when viewing the fearful faces than faces with other facial expressions (Geday, Gjedde, Boldsen, & Kupers, 2003; Vuilleumier, Armony, Driver, & Dolan, 2001) and that the VWFA was more activated by Kanji than Kana words (Horie, Yamasaki, Okamoto, Kan, et al., 2012). Fearful face photographs from eight individuals (four females) from the ATR face database (ATR Promotions, Inc.) were used, along with nine object photographs (e.g., shoes, house, telephone). All photographs were grayscale, sized 287 × 367 pixels (visual angle of 4° horizontally × 5.6° vertically). For the word stimuli, Japanese Kanji images were divided into four blocks per character, and each block was rotated, reversed, or shuffled randomly before rejoining the blocks, to make the scrambled‐word (SC) stimuli (Horie, Yamasaki, Okamoto, Nakashima, et al., 2012). Thirty early‐learned Kanji were chosen from the words learnt in the first and second grades in elementary school. Because they were familiar and easier than late‐learned Kanji (Horie, Yamasaki, Okamoto, Nakashima, et al., 2012), we increased the number of Kanji to avoid the repetition effect (Doyle & Rugg, 1998), and nine types of scrambled‐word stimuli were prepared (174 × 367 pixels; visual angle of 2.5° horizontally × 5.6° vertically for each two characters). The mean luminance and contrast were controlled by normalizing in each condition (luminance 50 cd/m^2^, contrast 80%) using MATLAB ver.7.4 (The MathWorks Inc.). The stimuli were presented either in the LVH or RVH, with the inner edge of the stimuli 2.5° horizontally from the fixation cross (Figure 1b). The viewing distance was 114 cm for binocular. For a pattern mask, a 1,024 × 768‐pixel noise pattern was generated with Adobe Photoshop 7.0. A (Adobe Inc.).

The experiments were conducted in a dimly lit, electrically shielded room, and participants sat on a comfortable chair. A 17‐inch CRT monitor (SONY Trinitron Multiscan G220) with a refresh rate of 60 Hz was used for the stimulus presentation. The stimuli were generated using Presentation software (Neurobehavioral Systems). The stimuli were followed by presentation of the central fixation cross on pattern masks for 500 ms (Figure 1c). Stimuli were presented with 2 different durations: subliminal (17 ms) and supraliminal (300 ms). The stimulus durations were chosen based on our previous ERP study (Mitsudo et al., 2011). Participants were instructed to respond by clicking a mouse as quickly as possible when the fixation cross changed color.

The experiments were carried out across 4 nonconsecutive days, 1 day each under of the four experimental conditions: the subliminal face/object, subliminal Kanji/SC word, supraliminal face/object, and supraliminal Kanji/SC word conditions. The conditions were tested on different days because each experiment took 1.5–2 hr. We used sensor net electrodes, with which head size was always measured and adjusted for each participant. To avoid repetition effects, the subliminal experiments were first performed followed by the supraliminal experiments and were counterbalanced by stimulus type across participants. All participants completed 2,400 trials [4 types of stimuli (face, object, word, SC) × 2 visual fields (left, right) × 2 presentation times (subliminal, supraliminal) × 15 sessions each × 10 trials each]. As an example, the participants were randomly presented with 40 trials/session, consisting of 10 trials of LVH‐face stimuli, 10 trials of LVH‐object stimuli, 10 trials of RVH‐face stimuli, and 10 trials of RVH‐object stimuli in the face recognition experiment. This procedure was repeated 15 times on each experiment day.

### 2.3 ERP recording and analysis

ERPs were recorded with a high‐density 128‐channel EEG system (NetAmps 200, Electrical Geodesics Inc.). ERP data were obtained with a vertex electrode (Cz) as the reference. The data were band‐pass filtered between 0.01 and 400 Hz and digitized at a sampling rate of 1,000 Hz. ERPs were processed offline using Net Station 4.2 software (Electrical Geodesics). The data were filtered using a 0.3–30‐Hz band‐pass filter and segmented from 100 ms before to 800 ms after the stimulus onset. Trials were rejected automatically if the amplitude exceeded 140 µV in any electrode, or if they contained more than 10 bad channels (in excess of 55 µV) as a result of eye movements. In the remaining trials, data from bad channels were interpolated from the remaining channels. Data were then re‐referenced to the average of the two electrodes closest to the tip of the nose (Horie, Yamasaki, Okamoto, Nakashima, et al., 2012). Data from at least 100 trials were averaged for each participant in each condition, and the baselines were corrected using the interval from 100 to 0 ms before stimulus onset.

**FIGURE 2:**
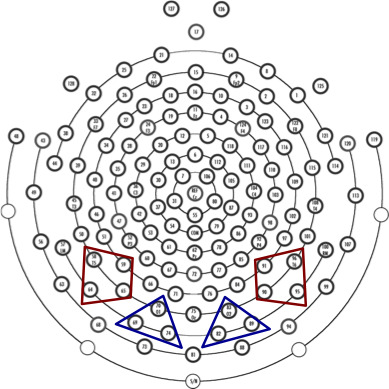
Regions of interest (ROIs) in a 128‐channel high‐density EEG system. We chose each ROI to select the left or right occipital area for P100 (blue triangles) and the left or right occipitotemporal area for N170 (red trapezoids)

Regions of interest (ROIs) were determined based on the topographic distribution of each component. The ROIs for LH‐P100s consisted of O1 with two adjacent electrodes, whereas those for RH‐P100s included O2 with two adjacent electrodes (Figure 2). Similarly, T5, with three adjacent electrodes, was selected for LH‐N170s, and RH‐N170s included T6, with three adjacent electrodes. These N170 components originate from fusiform gyrus, including the FFA and VWFA (Deffke et al., 2007; Horie, Yamasaki, Okamoto, Kan, et al., 2012; Horie, Yamasaki, Okamoto, Nakashima, et al., 2012; Yamasaki et al., 2016). The P100 was defined as the maximum positivity within an 80‐ to 130‐ms latency window (Mitsudo et al., 2011). N170 was defined as the maximum negativity within a 150‐ to 250‐ms latency window (Horie, Yamasaki, Okamoto, Nakashima, et al., 2012). Latencies and peak amplitudes from baseline of each ERP component were measured.

### 2.4 Statistical analysis

We adopted a three‐way analysis of variance (ANOVA) with repeated measures, to determine how the ERP responses were affected by three factors in the subliminal and supraliminal conditions for face/object and word/SC, respectively: stimulus type—face or object, word or SC word; visual hemifield—RVH or LVH; and hemisphere—LH and RH, since visual processing are different between face and word recognition, and between subliminal and supraliminal conditions. Peak amplitudes and latencies of P100 and N170 in the face and word experiments were analyzed.

All statistical analyses were performed using the Statistical Package for Social Sciences Version 22 (IBM Corp.), and *p* < .05 was regarded as statistically significant. Post hoc analyses were conducted for multiple comparisons using Tukey's test.

## 3 RESULTS

The mean numbers of epochs on which the analyses were performed are shown in Table 1. There were no statistically significant differences in the numbers of epochs among the conditions (subliminal face/object condition, subliminal word/SC condition, supraliminal face/object condition, and supraliminal word/SC condition, *p* = .59), and visual hemifield stimulations (LVH‐face, LVH‐object, LVH‐word, LVH‐SC, RVH‐face, RVH‐object, RVH‐word, and RVH‐SC, *p* = .1).

**TABLE 1:** The numbers of epochs/condition (mean ± *SD*)

	VH	Stimuli	Epochs
Subliminal	LVH	Face	127.1 ± 16.5
		Object	125.6 ± 19.0
	RVH	Face	127.6 ± 17.7
		Object	126.5 ± 16.2
	LVH	Word	132.7 ± 11.6
		SC	133.3 ± 11.5
	RVH	Word	133.4 ± 11.2
		SC	132.9 ± 10.1
Supraliminal	LVH	Face	136.3 ± 6.8
		Object	135.3 ± 8.7
	RVH	Face	135.7 ± 8.4
		Object	134.8 ± 10.3
	LVH	Word	133.3 ± 10.0
		SC	134.8 ± 9.9
	RVH	Word	132.2 ± 11.6
		SC	134.4 ± 9.9

Abbreviations: LVH, left visual field stimulation; RVH, right visual field stimulation; SC, scrambled words; VH, visual hemifield.

### 3.1 Subliminal condition

#### 3.1.1 Face‐ and word‐P100s

**FIGURE 3:**
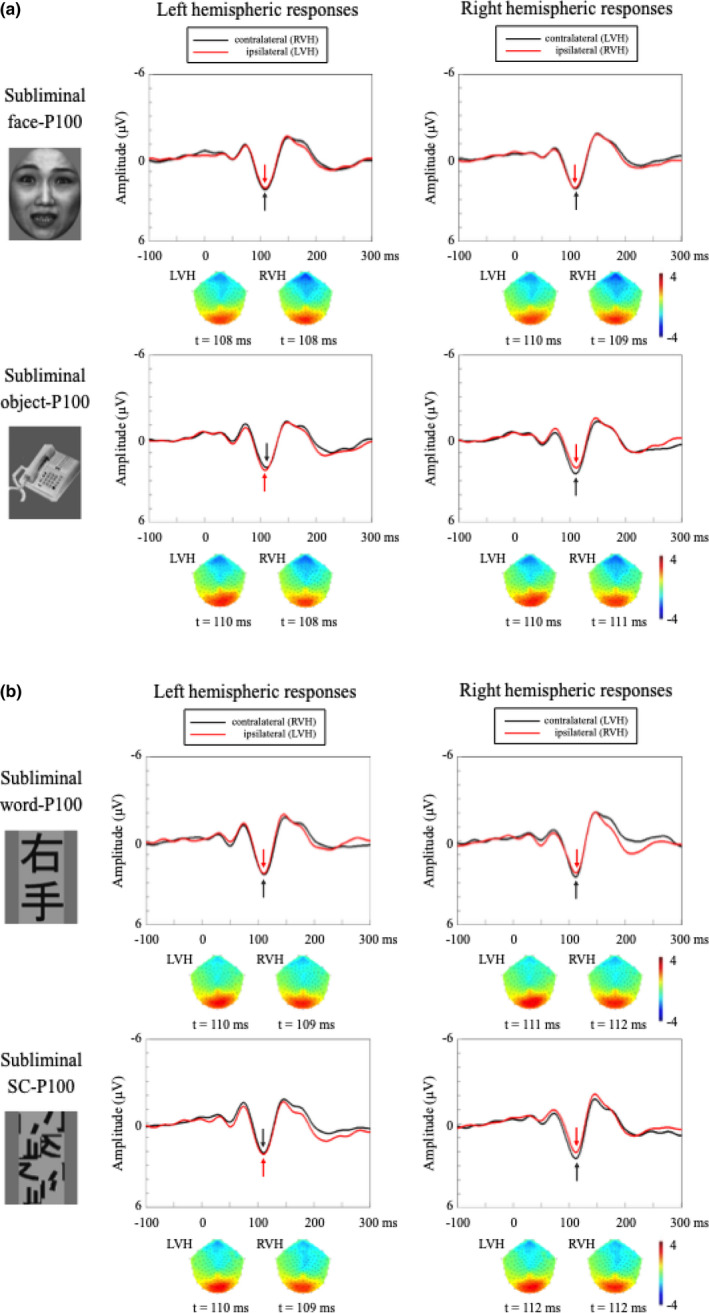
Grand‐averaged waveforms of P100 in response to the four visual stimuli in each hemifield in the subliminal condition. Black arrows indicate the peak responses with contralateral hemifield stimulation, while red arrows denote the peak responses with ipsilateral hemifield stimulation. (a) Face‐P100 in LH is at upper left, face‐P100 in RH is at upper right, object‐P100 in LH is at lower left, and object‐P100 in RH is at lower right. No obvious latency difference was found between contralateral and ipsilateral P100s. There was also no significant difference in amplitudes between face‐P100s and object‐P100s, regardless of hemisphere (left or right) and visual field (contralateral or ipsilateral). Scalp topographies were calculated at the peak of the P100 in each ROI using MATLAB, which showed the symmetrical distribution of the face‐P100 and object‐P100. (b) Word‐P100 in LH is at upper left, word‐P100 in RH is at upper right, SC‐P100 in LH is at lower left, and SC‐P100 in RH is at lower right. Like the behaviors of face‐ and object‐P100s, there was no significant difference in the amplitudes of word‐P100s and SC word‐P100s, regardless of hemisphere and visual field. Scalp topographies show the symmetrical distribution of the word‐ and SC‐P100s. LH, left hemisphere; LVH, left visual hemifield; RH, right hemisphere; ROI, region of interest; RVH, right visual hemifield; SC, scrambled

Grand‐averaged waveforms in response to the face/object and word/SC stimuli, and their scalp topographies in the occipital area, are shown in Figure 3a,b, respectively. The corresponding amplitudes and latencies of the P100s are summarized in Tables 2 and 3. The face/object‐P100s and word/SC‐P100s were evident in the occipital area without significant lateralization (Figure 3a,b). Accordingly, the three‐way ANOVA did not show significant main effects and interactions in the P100 amplitudes of the face/object and word/SC stimuli (Tables 4 and 5). Thus, no significant lateralization (LH or RH), contralateral predominance (contralateral or ipsilateral), or stimulus specificity (face or object, word or SC) was found under the subliminal condition.

**TABLE 2:** ERP results of the face experiment (mean ± *SE*)

	Left hemisphere		Right hemisphere	
	Contralateral (RVH)	Ipsilateral (LVH)	Contralateral (LVH)	Ipsilateral (RVH)
Amplitude (µV)				
Subliminal P100				
Face	3.5 ± 0.8	3.3 ± 0.6	3.5 ± 0.5	3.3 ± 0.7
Object	3.3 ± 0.5	3.3 ± 0.6	3.8 ± 0.5	3.1 ± 0.4
Supraliminal P100				
Face	3.3 ± 0.6	3.0 ± 0.6	3.7 ± 0.7	2.9 ± 0.5
Object	3.5 ± 0.7	2.9 ± 0.6	3.7 ± 0.7	3.2 ± 0.6
Supraliminal N170				
Face	−4.0 ± 0.5	−4.3 ± 0.6	−6.7 ± 0.6	−5.3 ± 0.7
Object	−2.6 ± 0.5	−2.8 ± 0.6	−3.1 ± 0.6	−3.9 ± 0.7
Latency (ms)				
Subliminal P100				
Face	111.4 ± 2.3	111.6 ± 2.2	112.0 ± 2.2	111.6 ± 2.4
Object	112.6 ± 2.2	110.9 ± 2.2	111.9 ± 2.6	112.9 ± 2.2
Supraliminal P100				
Face	107.9 ± 1.5	106.6 ± 1.8	107.4 ± 1.8	107.1 ± 1.6
Object	108.2 ± 1.6	108.2 ± 1.9	109.0 ± 1.9	107.7 ± 1.5
Supraliminal N170				
Face	170.9 ± 3.8	187.8 ± 4.7	169.3 ± 3.0	193.1 ± 4.1
Object	170.0 ± 5.3	177.2 ± 6.7	168.6 ± 3.5	175.2 ± 6.7

Abbreviations: LVH, left visual field stimulation; RVH, right visual field stimulation.

**TABLE 3:** ERP results of the word experiment (mean ± *SE*)

	Left hemisphere		Right hemisphere	
	Contralateral (RVH)	Ipsilateral (LVH)	Contralateral (LVH)	Ipsilateral (RVH)
Amplitude (µV)				
Subliminal P100				
Word	2.9 ± 0.4	3.0 ± 0.4	2.8 ± 0.4	3.0 ± 0.4
SC	2.7 ± 0.4	3.1 ± 0.5	2.8 ± 0.4	2.8 ± 0.4
Supraliminal P100				
Word	3.1 ± 0.5	2.4 ± 0.3	2.9 ± 0.5	2.4 ± 0.4
SC	2.8 ± 0.6	2.5 ± 0.5	2.9 ± 0.5	2.2 ± 0.5
Supraliminal N170				
Word	−4.9 ± 0.5	−4.6 ± 0.5	−5.1 ± 0.6	−4.3 ± 0.6
SC	−4.9 ± 0.7	−4.3 ± 0.5	−4.7 ± 0.6	−4.5 ± 0.5
Latency (ms)				
Subliminal P100				
Word	109.9 ± 2.0	109.2 ± 2.0	108.6 ± 1.9	109.8 ± 1.9
SC	107.7 ± 2.4	110.1 ± 2.2	110.3 ± 2.1	109.9 ± 1.9
Supraliminal P100				
Word	107.9 ± 1.3	106.0 ± 1.6	107.3 ± 1.7	107.5 ± 1.4
SC	108.0 ± 1.4	106.1 ± 1.5	108.1 ± 1.7	107.9 ± 1.5
Supraliminal N170				
Word	184.1 ± 5.1	200.4 ± 5.0	179.5 ± 5.9	211.3 ± 5.7
SC	184.7 ± 4.9	201.3 ± 5.3	184.4 ± 5.7	216.8 ± 5.6

Abbreviations: LVH, left visual field stimulation; RVH, right visual field stimulation; SC, scrambled words.

**TABLE 4:** Statistical results for the face experiment

	*df*	*F*	*p*	$\eta_{p}^{2}$
Subliminal				
P100 amplitude				
Visual field	1	2.38	.13	.022
Hemisphere	1	0.39	.53	.004
Stimulus type × Visual field	1	0.23	.63	.002
Stimulus type × Hemisphere	1	0.11	.74	.001
Visual field × Hemisphere	1	0.64	.43	.006
Stimulus type × Visual field × Hemisphere	1	1.16	.26	.011
Supraliminal				
P100 amplitude				
Stimulus type	1	0.55	.46	.005
Visual field	1	16.1	.0001**	.13
Hemisphere	1	1.37	.25	.013
Stimulus type × Visual field	1	0.018	.89	<.0001
Stimulus type × Hemisphere	1	0.16	.40	.002
Visual field × Hemisphere	1	0.73	.69	.007
Stimulus type × Visual field × Hemisphere	1	1.14	.29	.011
N170 amplitude				
Stimulus type	1	70.7	<.0001**	.45
Visual field	1	2.4	.13	.026
Hemisphere	1	27.6	<.0001**	.24
Stimulus type × Visual field	1	0.5	.47	.006
Stimulus type × Hemisphere	1	2.8	.09	.032
Visual field × Hemisphere	1	0.6	.44	.007
Stimulus type × Visual field × Hemisphere	1	6.3	.014*	.068
N170 latency				
Stimulus type	1	6.8	.011*	.072
Visual field	1	26.0	<.0001**	.23
Hemisphere	1	0.044	.83	.001
Stimulus type × Visual field	1	5.4	.023*	.058
Stimulus type × Hemisphere	1	0.32	.57	.004
Visual field × Hemisphere	1	0.14	.71	.002
Stimulus type × Visual field × Hemisphere	1	0.38	.54	.004

Abbreviation: *df*, degree of freedom.

* *p* < .05.

** *p* < .01

**TABLE 5:** Statistical results for the word experiment

	*df*	*F*	*p*	$\eta_{p}^{2}$
Subliminal				
P100 amplitude				
Stimulus type	1	0.88	.35	.008
Visual field	1	3.1	.080	.029
Hemisphere	1	0.21	.65	.002
Stimulus type × Visual field	1	0.10	.75	.001
Stimulus type × Hemisphere	1	0.041	.84	<.0001
Visual field × Hemisphere	1	0.73	.40	.007
Stimulus type × Visual field × Hemisphere	1	1.4	.24	.013
Supraliminal				
P100 amplitude				
Stimulus type	1	0.64	.43	.006
Visual field	1	14.0	<.0001**	.12
Hemisphere	1	0.48	.49	.005
Stimulus type × Visual field	1	0.16	.69	.002
Stimulus type × Hemisphere	1	0.017	.90	<.0001
Visual field × Hemisphere	1	0.02	.89	<.0001
Stimulus type × Visual field × Hemisphere	1	1.7	.19	.016
N170 amplitude				
Stimulus type	1	0.006	.94	<.0001
Visual field	1	2.7	.10	.031
Hemisphere	1	0.32	.58	.004
Stimulus type × Visual field	1	0.001	.98	<.0001
Stimulus type × Hemisphere	1	0.010	.92	<.0001
Visual field × Hemisphere	1	0.32	.57	.004
Stimulus type × Visual field × Hemisphere	1	1.1	.29	.013
N170 latency				
Stimulus type	1	0.72	.40	.008
Visual field	1	60.9	<.0001**	.417
Hemisphere	1	1.8	.18	.021
Stimulus type × Visual field	1	0.005	.95	<.0001
Stimulus type × Hemisphere	1	0.34	.56	.004
Visual field × Hemisphere	1	5.1	.027*	.056
Stimulus type × Visual field × Hemisphere	1	0.082	.775	.001

Abbreviation: *df*, degree of freedom

* *p* < .05.

** *p* < .01

#### 3.1.2 Face‐ and word‐N170s

No face‐ or word‐N170s were elicited, regardless of the stimulus type or stimulus visual field. Thus, we did not perform further analysis of the N170 component under the subliminal condition.

### 3.2 Supraliminal condition

#### 3.2.1 Face‐ and word‐P100s

**FIGURE 4:**
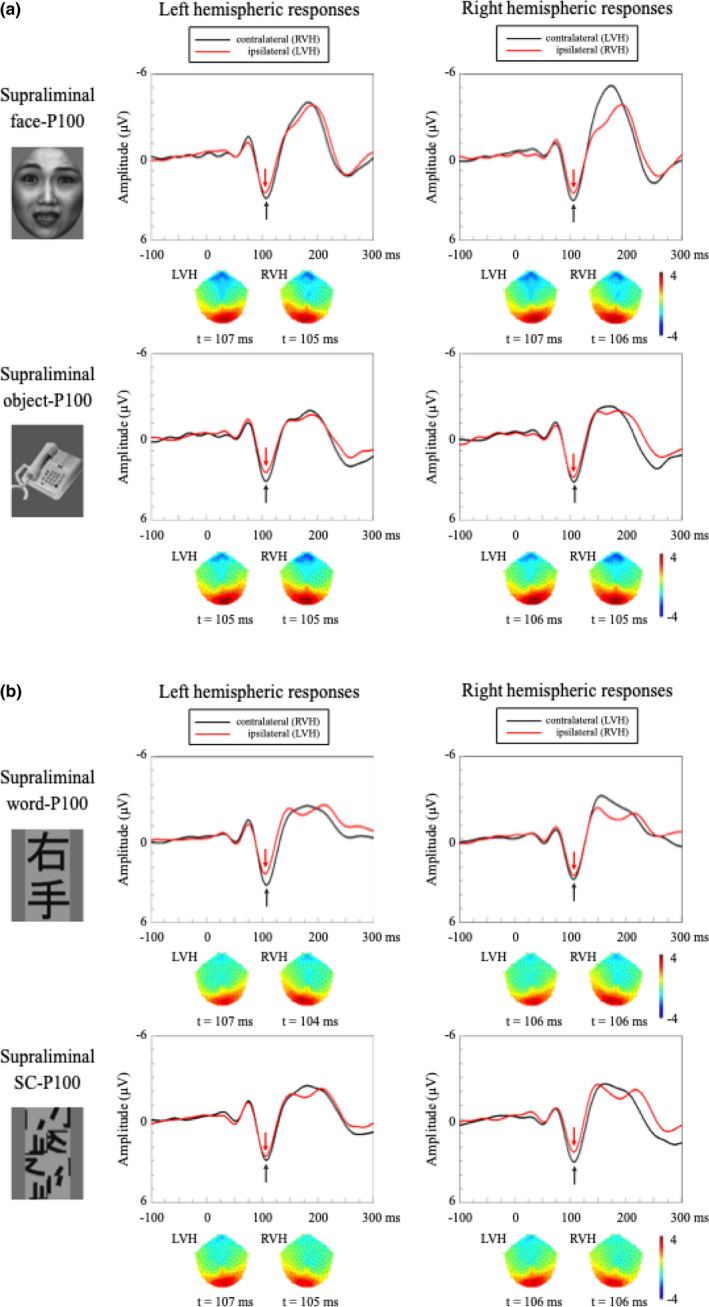
Grand‐averaged P100 waveforms in response to the four visual stimuli in each hemifield, in the supraliminal condition. Black arrows indicate the peak responses with contralateral hemifield stimulation, while red arrows denote the peak responses with ipsilateral hemifield stimulation. (a) Face‐P100 in LH is at upper left, face‐P100 in RH is at upper right, object‐P100 in LH is at lower left, and object‐P100 in RH is at lower right. Unlike the subliminal condition (see Figure 3), the contralateral P100 amplitudes were larger than the ipsilateral P100s, irrespective of hemisphere (left or right) and stimuli (face or object). Scalp topographies also showed the asymmetrical distribution of the P100 amplitudes. However, no apparent latency difference between contralateral‐ and ipsilateral‐P100s was present. (b) Word‐P100 in LH is at upper left, word‐P100 in RH is at upper right, SC‐P100 in LH is at lower left, and SC‐P100 in RH is at lower right. Like the behaviors of face‐ and object‐P100s, the contralateral P100 amplitudes were larger than ipsilateral ones in the word and SC conditions, irrespective of hemisphere and stimuli. Scalp topographies also showed the asymmetrical distribution of the P100 amplitudes

Grand‐averaged waveforms in response to the face/object and word/SC stimuli, and their scalp topographies in the occipital area, are shown in Figure 4a,b, respectively. Tables 2 and 3 list the amplitudes and latencies of the P100s for face/object and word/SC stimuli. P100s for the face/object and word/SC stimuli were evident in the occipital area. In contrast to the subliminal condition, the P100 amplitudes showed contralateral predominance for the visual field, irrespective of the stimulus type. This finding was confirmed by the three‐way ANOVA (Tables 4 and 5). There was a significant main effect of visual field on the face‐/object‐P100s (contralateral P100 [3.6 ± 0.6 µV] > ipsilateral P100 [3.0 ± 0.6 µV], *F* (1,105) = 16.1, *p* < .001, MSe = 0.59, $\eta_{p}^{2}$ = .13) and the word/SC‐P100s (contralateral P100 [2.9 ± 0.5 µV] > ipsilateral P100 [2.4 ± 0.5 µV], *F* (1,105) = 13.9, *p* < .001, MSe = 0.68, $\eta_{p}^{2}$ = .12). No other main effects (stimulus type or hemisphere) or interactions were observed. In terms of latency, there was no significant main effect or interaction in any stimulus condition (Tables 4 and 5).

#### 3.2.2 Face‐N170

**FIGURE 5:**
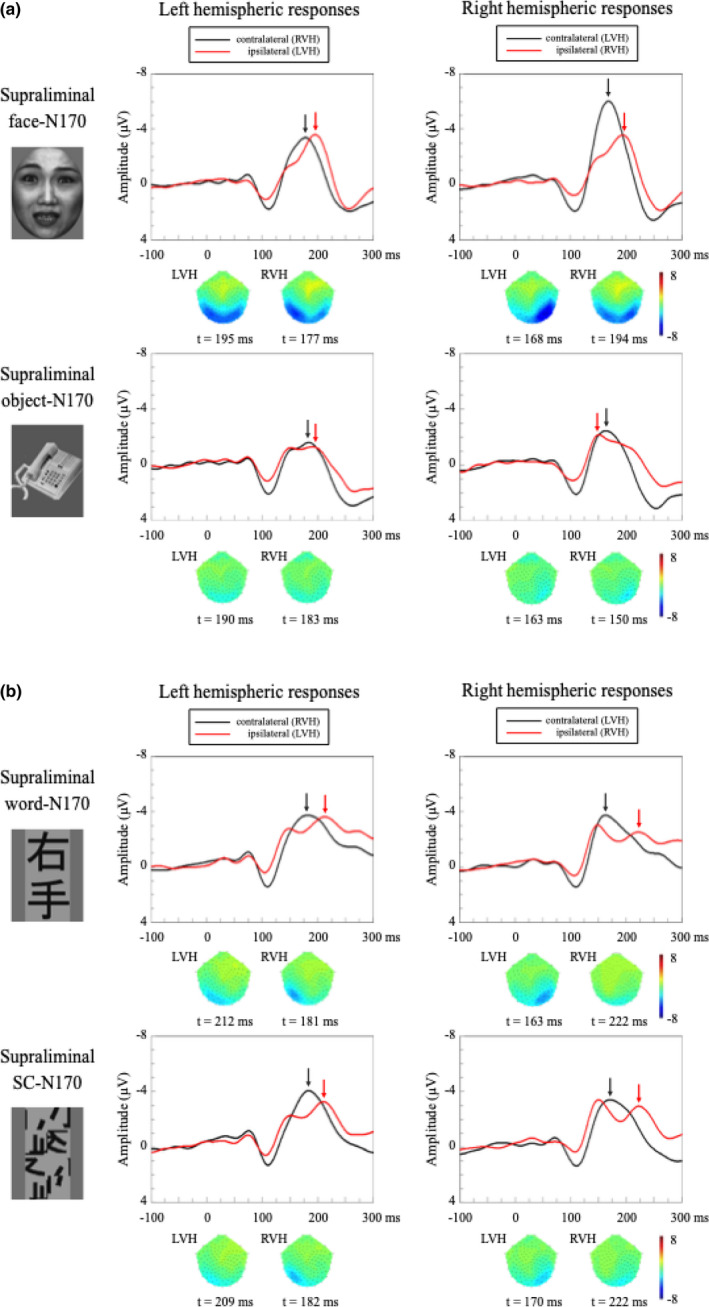
Grand‐averaged waveforms of N170 in response to the four visual stimuli in each hemifield in the supraliminal condition. Black arrows indicate the peak responses with contralateral hemifield stimulation, while red arrows denote the peak responses with ipsilateral hemifield stimulation. (a) Face‐N170 in LH is at upper left, face‐N170 in RH is at upper right, object‐N170 in LH is at lower left, and object‐N170 in RH is at lower right. Face‐ and word‐N170s were evident over the occipitotemporal area. The face‐N170 was larger than the object one. Note that the amplitude of the face‐N170 in the right hemisphere was the largest among the four stimulus conditions. Such hemispheric lateralization was not seen in the object‐N170. (b) Word‐N170 in LH is at upper left, word‐N170 in RH is at upper right, SC‐N170 in LH is at lower left, and SC‐N170 in RH is at lower right. Word‐ and SC‐N170s were evident over the occipitotemporal area. Unlike the face‐ and object‐N170s, there was no significant difference in the N170 amplitudes for the two types of word stimuli, regardless of hemisphere (left or right) and visual field (contralateral or ipsilateral)

Figure 5a shows the grand‐averaged waveforms in response to the face/object stimuli and their scalp topographies in the occipitotemporal area. The amplitudes and latencies of the N170s for the face/object stimuli are also summarized (Tables 2 and 3). The face‐N170 was clearly identifiable, in contrast to that in the subliminal condition (Figures 3a and 5a, upper). The object‐N170 was less apparent, compared with the face‐N170 (Figure 5a, lower). The three‐way ANOVA showed significant main effects for stimulus type (face‐N170 (−5.0 ± 0.5 µV) > object‐N170 (−2.8 ± 0.5 µV), *F* (1,87) = 70.7, *p* < .001, MSe = 1.80, $\eta_{p}^{2}$ = .45) and hemisphere (LH N170 (−3.2 ± 0.5 µV) < RH N170 (−4.6 ± 0.5 µV), *F* (1,87) = 27.6, *p* < .001, MSe = 1.80, $\eta_{p}^{2}$ = .24; Table 4). A significant interaction was also found among stimulus type, visual hemifield, and hemisphere (*F* (1,87) = 6.3, *p* = .014, MSe = 1.80, $\eta_{p}^{2}$ = .068; Table 4). These findings indicate that the amplitude of the face‐N170 (−5.0 ± 0.5 µV) was significantly larger than that of the object‐N170 (−2.8 ± 0.5 µV, *p* < .001) and that the face‐N170 in the RH (−5.9 ± 0.5 µV) was larger than that in the LH (−4.1 ± 0.5 µV, *p* < .001; Figure 5a). There were no other main effects or interactions.

Regarding the N170 latency, significant main effects of stimulus type (*F* (1,87) = 6.8, *p* = .011, MSe = 170.3, $\eta_{p}^{2}$ = .072) and visual field (*F* (1,87) = 26.0, *p* < .001, MSe = 170.3, $\eta_{p}^{2}$ = .23) were found (Table 4), indicating that the face‐N170 latency (179.8 ± 3.3 ms) was longer than that of the object‐N170 (172.9 ± 3.5 ms; Tables 2 and 4, *p* < .001) and that the ipsilateral N170 latency (183.0 ± 3.5 ms) was longer than the contralateral one (169.7 ± 3.3 ms; Tables 2 and 4, *p* < .001). Moreover, a significant interaction between stimulus type and visual field was found (contralateral face‐N170 (170.1 ± 3.7 ms), ipsilateral face‐N170 (176.5 ± 4.2 ms), contralateral object‐N170 (169.3 ± 3.7 ms), ipsilateral object‐N170 (170.1 ± 3.7 ms), and *F* (1,87) = 5.4, *p* = .023, MSe = 170.3, $\eta_{p}^{2}$ = .058).

#### 3.2.3 Word‐N170

Figure 5b shows the grand‐averaged waveforms in response to the word/SC stimuli and their scalp topographies in the occipitotemporal area. Tables 2 and 3 summarize the amplitudes and latencies of the N170s for word/SC stimuli. The word‐N170 was clearly identifiable, contrary to that in the subliminal condition (Figure 5b, upper). The SC‐N170 was less clearly delineated than the word‐N170 (Figure 5b, lower). For the N170 amplitudes, the three‐way ANOVA showed that there were no significant main effects and interactions in the word experiment (Table 5). Thus, unlike the face‐N170, the word‐N170 showed no contralateral predominance with stimulus specificity.

For the N170 latency, a significant main effect of visual field (contralateral N170 (183.1 ± 3.9 ms) < ipsilateral N170 (207.6 ± 4.0 ms)*, F* (1,85) = 60.9, *p* < .001, MSe = 254.4, $\eta_{p}^{2}$ = .42) and an interaction between visual field and hemisphere were observed (Table 5, *F* (1,85) = 5.1, *p* = .027, MSe = 254.4, $\eta_{p}^{2}$ = .056). This indicates that the latency of the ipsilateral N170 was longer than that of the contralateral one (LH contralateral N170 (184.5 ± 4.3 ms), RH contralateral N170 (181.7 ± 4.6 ms) < LH ipsilateral N170 (201.8 ± 4.5 ms), and RH ipsilateral N170 (213.4 ± 4.8 ms), *p* < .001). No main effects of stimulus type or hemisphere were found.

## 4 DISCUSSION

Our major findings are summarized as follows. First, the contralateral P100 amplitude was greater than the ipsilateral one, for all stimulus types, in the supraliminal condition, but this effect was not observed in the subliminal condition. Second, both the face‐ and word‐N170 responses were elicited in the supraliminal condition, but not in the subliminal. Third, in the supraliminal condition, the face‐N170 amplitude was significantly larger than the object‐N170, and hemispheric specialization for face recognition was found regardless of the stimulus visual hemifield. On the contrary, the word‐N170 amplitude was not significantly affected by the stimulus type, stimulus visual field, or hemisphere. Taken together, these findings suggest that both P100 and N170 were differentially affected by the nature of the visual stimuli, depending on the stimulus visual field and presentation duration.

### 4.1 Visual awareness is necessary for face and word recognition

Contralateral predominance of the P100 amplitude, irrespective of the stimulus type, was not observed in the subliminal condition. This suggests that visual awareness is essential for spatial information processing in V1. P100 amplitude correlated with subjective visibility (“seen” or “unseen”) and selective attention in the study by Mathewson, Gratton, Fabiani, Beck, and Ro (2009). A previous review also stressed the earlier finding that conscious perception correlated with enhanced P100 amplitudes, when compared with conditions where the same stimulus was not consciously perceived (Railo, Koivisto, & Revonsuo, 2011). It is assumed that the P100 correlates of consciousness reflect the earliest feedback interactions between early visual cortical areas (Boehler, Schoenfeld, Heinze, & Hopf, 2008). Consistent with this idea, V1 cannot work as a feedback processor because of a lack of awareness of the subliminal visual hemifield stimulation in this study.

In our study, there were no significant differences in P100 amplitudes across the stimulus types (face/object, word/SC) and experimental conditions (stimulus visual hemifield, hemisphere) in the subliminal condition. This finding is the opposite of that in our previous ERP study using full‐field face images. In that study, the P100 to the face stimuli was larger than that to the object stimuli in the subliminal condition (Mitsudo et al., 2011). Subliminal face processing depends on LSF information (Itier & Taylor, 2004). Thus, it is likely that faces are identified through holistic visual processing using coarse visual cues with a brief presentation. However, in our experiment, the processing capability for LSF information was decreased by using hemifield stimulation. In the case of subliminal visual hemifield stimulation, it may depart from the preferred SFs to identify faces by both greater eccentricities, with visual hemifield stimuli, and the masking paradigm (Lu et al., 2018). Thus, no P100 amplitude differences across the experimental conditions were seen in the present study.

Furthermore, no difference in the P100 amplitudes between the Kanji and SC words was observed under the subliminal condition. As far as we know, no study has been performed with both full‐field and hemifield stimulation using Kanji and SC words. Therefore, it is necessary in the future to study the effect of visual lack of awareness on Kanji and SC words, with full‐field stimulation, to test whether this finding is specific to the hemifield stimulation.

Neither face‐ nor word‐N170s were evoked in the subliminal condition. This suggests that neural activation occurred in neither the FFA nor VWFA. In our previous study (Mitsudo et al., 2011), the face‐N170 amplitude from the subliminal (invisible) stimuli was less marked than that from the visible stimuli. Likewise, Dehaene et al. (2001) showed that VWFA was less activated by the unconscious word than by the conscious. Together with these findings, it appears that awareness is essential for evoking both the face‐ and word‐N170s.

### 4.2 Importance of stimulus physical features for V1 activation

Unlike the subliminal condition, contralateral predominance of the face‐ and word‐P100s was observed in V1 in the supraliminal condition (Figure 4). Whenever the visual stimuli presented in the hemifield were vividly recognized, a P100 peak was clearly evoked, irrespective of the stimulus type. This finding is consistent with our previous study with full‐field visual stimulation, wherein the amplitude and latency of the face‐P100s were not significantly different from those of the object‐P100s (Mitsudo et al., 2011). Generally, V1 activation is affected by stimulus parameters such as contrast, luminance, size, and location (Tobimatsu & Celesia, 2006). Even in this hemifield study, the P100 response was unaffected by the physical characteristics of the visual stimuli because we carefully matched the stimulus parameters as much as possible.

Regarding the word stimuli, our previous ERP study with supraliminal visual full‐field stimulation demonstrated larger P100s to SC stimuli than to Kanji word stimuli (Horie, Yamasaki, Okamoto, Nakashima, et al., 2012). That may be caused by the difference in SF information processing (i.e., the SC words were composed of multidirectional components, whereas the Kanji words were composed of horizontal and vertical components). Hence, the SF‐processing ability in V1 might have been reduced in the present study so that the word‐ and SC‐P100 amplitude differences could not be observed.

### 4.3 Right FFA is specialized for face recognition

In contrast to the P100 amplitudes, contralateral preference was not observed in the N170 amplitudes in the supraliminal condition, and the right FFA was activated most for the face‐N170. However, no hemispheric specialization for object‐N170 amplitudes was found, as opposed to that observed in a study by Rossion et al. (2003). It has been reported that the contralateral preference decreases at higher levels of the visual ventral stream (Grill‐Spector et al., 1998), whereas the responses for stimulus properties (e.g., faces) increase (Grill‐Spector & Malach, 2004). In other words, contralateral predominance decreases from V1 to the FFA. However, that does not necessarily mean that the contralateral predominance is totally lost at higher visual‐processing levels. In accordance with this idea, a previous functional magnetic resonance imaging (fMRI) study showed a weaker contralateral preference in the FFA than in the lateral occipital regions (Hemond et al., 2007). In our ERP study, contralateral predominance was not found in the FFA, although the face‐P100 showed contralateral predominance. Discrepant results between ours and those of Hemond et al. (2007) are probably due to the stimulus position and size. They presented their 8° × 8° face stimuli 1° away from the fixation spot. Ours were 2.4° × 5.6° and presented 2.5° away from the fixation cross, so more peripheral field was stimulated.

Perceptual capacity depends on where stimuli are located in the visual field, because of the sparse distribution of receptors and the neural structures of the visual cortices. It has been shown that in FFA nearly all neural resources are dedicated to the central (~7°) portion of the visual field (Kay, Weiner, & Grill‐Spector, 2015). In addition, with face stimuli, the N170 declines if presented a few degrees away from the central fixation (Eimer, 2000). Therefore, the present result is interesting in that only the right FFA showed the largest response to faces irrespective of the visual field. Similarly, Kovacs, Knakker, Hermann, Kovacs, and Vidnyanszky (2017) reported that the face‐N170 showed right hemispheric specialization without contralateral predominance, regardless of RVH or LVH. This is consistent with the right hemisphere being specialized for face recognition (Davies‐Thompson et al., 2016; Kanwisher, McDermott, et al., 1997). Because socially important information, such as facial emotions, is not always perceived in the central visual field, humans might be able to discriminate faces presented in the periphery better than they can words. This raises the possibility that holistic processing for faces may be also involved in recognition of faces presented in the peripheral visual field (Farah, Wilson, Drain, & Tanaka, 1998; Jacques & Rossion, 2009; Rossion, 2008).

### 4.4 Left VWFA needs integrated spatial information of each visual field for word recognition

Unlike the face‐N170, the word‐N170 amplitudes did not show hemispheric specialization. Regardless of stimulus type, the ipsilateral N170 latencies were longer than contralateral ones. On the contrary, Cohen et al. (2002) found that the left VWFA was more activated by letters than by a checkerboard and that word stimuli in the RVH induced predominant responses in the LH. This property was also reported in a behavioral study (Selpien et al., 2015). Together with these findings, it is suggested that VWFA in the LH is modulated by visual hemifield stimuli for word recognition. In addition, our previous study showed that the LH word‐N170 from real words presented in the full field was significantly larger than that of the RH, but that was not the case for SC‐N170 (Horie, Yamasaki, Okamoto, Nakashima, et al., 2012). Another ERP study found that brain responses before ~200‐ms post‐stimulus‐onset distinguish words from pseudo‐words (Bentin et al., 1999; Mariol, Jacques, Schelstraete, & Rossion, 2008). These reports suggested that the word‐N170 component is sensitive to visual word form features.

Discrepant results between our study and previous studies may result from the intersubject variability of the ipsilateral N170 (Figure 5b). The word‐N170 peak was somewhat broadened in the grand‐averaged responses, unlike the face‐N170 peak. As an alternative interpretation, the discrepancy may be due to the differences between foveal and parafoveal neuronal responses. There might be insufficient local information to discriminate between the Kanji and SC words with increasing eccentricity of the hemifield stimuli. Theoretically, we may have failed to fully activate the foveal neurons in the present study, because of the stimulus eccentricity, thus evoking less VWFA activation for the Kanji and SC in the hemifield condition. Because the sensitivity for high SFs decreases with increasing eccentricity within the visual hemifield (Pointer & Hess, 1989), the present subjects might not have identified the differences between the Kanji and SC words in the parafovea. In other words, the LH specialization for visual word recognition might only be observed when stimuli are perceived at the fovea. Even in daily reading, it seems that we identify words more clearly in the foveal visual field than the parafoveal. Jordan, Fuggetta, Paterson, Kurtev, and Xu (2011) recorded the word‐N170 when presenting words in the foveal or parafoveal visual field; they found larger word‐N170 amplitudes from stimuli at the fovea. Therefore, it is likely that there is preferential word recognition in the foveal visual field and that LH specialization for words is modulated by specific SFs (i.e., RH: low SF, LH: high SF; Musel et al., 2013).

## 5 LIMITATIONS

Some limitations exist in the present study. First, we adopted fearful faces as the face stimuli to elicit larger ERP components in both the subliminal and supraliminal conditions. The N170 amplitudes are well known to be larger with fearful faces than with neutral faces in previous ERP studies (Blau, Maurer, Tottenham, & McCandliss, 2007; Jiang et al., 2009). Fearful faces are distinguished from other expressions during early visual processing (Zhang et al., 2014). Furthermore, in a proposed face‐processing model, both facial identity and expression are first encoded by a mechanism that is not completely separated within a single visual perceptual representation (Calder & Young, 2005). It was reported that there is not a strict correspondence between behavioral evidence and ERP components regarding hemispheric asymmetries for emotions (Prete, Capotosto, Zappasodi, & Tommasi, 2018). However, a recent study has reported hemispheric asymmetries in emotion processing (Wyczesany, Capotosto, Zappasodi, & Prete, 2018), and subliminal emotional LSF information affects early visual processing (Prete, Capotosto, Zappasodi, Laeng, & Tommasi, 2015). This does not exclude a possible effect of emotion on our results. It is also known that N170 is modulated by task‐related attention (i.e., face detection; Krolak‐Salmon, Fischer, Vighetto, & Mauguiere, 2001; Wronka & Walentowska, 2011), but this study did not test the effect of attention in the subliminal hemifield condition.

Second, visual stimuli were presented in either the RVH or LVH, but not in the central visual field (i.e., full‐field stimulation), to explore functional hemispheric specialization. Thus, our results in the subliminal face recognition were compared with our previous research based on full‐field stimulation (Mitsudo et al., 2011). However, we are not certain that this is analogous for subliminal word recognition. Therefore, future study is necessary to determine whether the subliminal effect depends on the central visual field in word recognition during early visual processing.

Finally, Japanese people use several character sets, such as Kanji and Hiragana, on a daily basis. It is necessary to investigate whether the Japanese word recognition system differs from language systems used in other countries. In particular, the dependence on the visual field and type of characters are matters of interest. Recent studies have reported gender differences, that right hemispheric specialization was only males in face recognition and left hemispheric specialization was only females in word recognition (Ji, Cao, & Xu, 2016). Further study should investigate the plastic changes in the brain function due to the environment, habits, and gender.

## 6 CONCLUSIONS

We systematically investigated ERP responses to face and word stimuli, using visual hemifield stimulation, in Japanese adults who routinely use Kanji words. Our results suggest that visual awareness is essential for face and word recognition. In supraliminal face and word recognition processing, contralateral predominance was found in V1. Consequently, the face recognition in the FFA showed right hemispheric specialization even with hemifield stimulation, but the VWFA did not show specialization. Taken together, our results provide electrophysiological evidence of hemispheric specialization in the fusiform gyrus, depending on the stimulus category, in the Japanese brain. Our study using hemifield stimulus presentation further demonstrates the robust right FFA for face recognition but not the left VWFA for word recognition.

## CONFLICT OF INTEREST

The authors declare that they have no competing interests.

## AUTHOR CONTRIBUTIONS

NT analyzed the data and wrote this manuscript. TM, TY, and KO engaged in manuscript writing and data analyses. EY and MT participated in data collection and revised the manuscript. ST engaged in the conception of this manuscript writing and revised the manuscript.

## Data Availability

The data that support the findings of this study are available from the corresponding author upon reasonable request.
